# Evaluation of Uterosacral Ligament Involvement in Deep Endometriosis by Transvaginal Ultrasonography

**DOI:** 10.3389/fphar.2019.00374

**Published:** 2019-04-11

**Authors:** Yujuan Zhang, Xiaojun Xiao, Fanhua Xu, Qi Lin, Jinfeng Xu, Bo Du

**Affiliations:** ^1^Department of Ultrasound, Shenzhen People’s Hospital, The Second Clinical Medical College of Jinan University, The First Affiliated Hospital of Southern University of Science and Technology, Shenzhen, China; ^2^Shenzhen Medical Ultrasound Engineering Center, Shenzhen, China; ^3^Department of Emergency, Shenzhen People’s Hospital, The Second Clinical Medical College of Jinan University, The First Affiliated Hospital of Southern University of Science and Technology, Shenzhen, China

**Keywords:** uterosacral ligament, transvaginal sonography, endometriosis, deep infiltrating endometriosis (DIE), diagnosis

## Abstract

This study was designed to conclude the ultrasonic characteristics of uterosacral ligament (USL) lesions involved by endometriosis and evaluated the value of transvaginal sonography (TVS) in diagnosing USL involvement in deep infiltrating endometriosis (DIE). A total of one hundred and eighteen patients with DIE were included in the study and underwent surgery. All these patients were evaluated by transvaginal ultrasound examination by one trained examiner. The gold standard for diagnosis was surgery and histopathology. 85 patients with USL endometriosis were confirmed by surgical pathology. 84 patients were diagnosed USL endometriosis by TVS and 81 of which were confirmed by the gold standard. The sensitivity, specificity, positive predictive value (PPV), negative predictive value (NPV), and accuracy of TVS for diagnosing USL endometriosis were 95.3, 90.9, 96.4, 88.2, and 94.1%, respectively. According to the ultrasound characteristics of USL endometriosis, we summarized four types: Type I. thickened and stiff lesions, Type II. local nodules, Type III. irregular striped lesions, and Type IV. mixed lesions. The conclusion of the study was that TVS was a convenient, accurate and first-line diagnostic technique for USL endometriosis and the USL lesions could be summarized into four types according to the ultrasound morphological changes.

## Introduction

Deep infiltrating endometriosis (DIE) was defined as an endometriotic lesion that infiltrates the peritoneum and penetrates into the retroperitoneal space or the wall of the pelvic organs to a depth of at least 5 mm (Koninckx and Martin, 1992). It occurs in 15–30% of patients with endometriosis (Yantiss et al., 2001). The most common involved location was uterosacral ligament (USL) (Chapron et al., 2003; Bazot et al., 2007). The involvement of USL may cause many clinical symptoms, such as chronic pelvic pain and deep dyspareunia (Hummelshoj et al., 2014). However, there was always a delay between the onset of the first symptoms and the clinical diagnosis of endometriosis and usually the interval was approximately 7–10 years (Matsuzaki et al., 2006; Hudelist et al., 2012) because of the low sensitivity of TVS for USL endometriosis (Bazot et al., 2004; Vimercati et al., 2012; Holland et al., 2013). The latest meta-analysis (Guerriero et al., 2015) for detection of USL endometriosis demonstrated that the overall pooled sensitivity of transvaginal sonography (TVS) was only 53% (95% confidence interval (CI), 35–70%). There were many reasons for the low diagnostic rate, such as the small space of posterior pelvic compartment and its complex structure, the diversity of ultrasound morphological characteristics and the differences of examiners’ experiences. The aims of this study were to assess the value of TVS for diagnosing USL endometriosis performed by an experienced examiner and summarize the ultrasound morphological features of USL endometriosis, so that the examiners could quickly identify the lesion of the USL and improve the diagnostic rate.

## Materials and Methods

### Ethics Statement

The Medical Science Ethics Committee of Shenzhen People’s Hospital approved this study (NO. 2018100). Each patient or an appropriate family member provided informed written consent to obtain clinical materials.

### Study Population

From October 2013 to October 2017, a total of 118 patients met the inclusion and exclusion criteria for the study. All patients were enrolled from the Shenzhen People’s Hospital.

### Inclusion and Exclusion Criteria

#### Inclusion Criteria

(1) Patients were diagnosed as DIE according to their clinical data. (2) Patients needed surgery treatment.

#### Exclusion Criteria

(1) Patients withdrawed from the study for personal reasons. (2) Patients who were pregnant while waiting for surgery. (3) Patients have not undergone surgery for any reasons.

### Imaging Techniques

All TVS scans were performed by one examiner who had received professional training. The examiner was blinded to physical examination and previous imaging examination results but was aware that the women were being evaluated for chronic pelvic pain and that endometriosis was suspected. All the TVS examinations were performed within 2 weeks of surgery.

All patients were examined in the lithotomy position using either a GE E8 (GE Healthcare Ultrasound, United States) or Philips IU22 (Philips IU22, United States) scanner equipped with 5–9-MHz or C10-3 transducer for transvaginal visualization.

### TVS Techniques

Transvaginal sonography examinations were performed with ultrasound transmission gel in the probe cover to create a stand-off to visualize the near-field area. In all patients, the uterus and ovaries were detected first to rule out adenomyosis and ovarian cysts, which are frequently associated with DIE (Somigliana et al., 2004; Chapron et al., 2009). Then, the transducer was withdrawn to the perineum and inserted into the vagina slowly to evaluate the vagina, rectovaginal septum, pouch of Douglas, USLs, bowel walls, etc., All of involved targets, especially painful sites, were evaluated in multiple scanning planes by rotating the transducer. The lesion size was measured in three orthogonal planes.

Uterosacral ligament involvement in DIE was best evaluated by placing the transvaginal probe in the posterior vaginal fornix at the midline in a sagittal plane and then sweeping the probe inferolaterally to the cervix.

The echogenicity, changes in shape, thickness, and size of the USLs were observed, described and recorded for analysis. The thickness of a “thickened” USL was measured in the transverse plane at the insertion of the ligament at the cervix.

### Statistical Analysis

The ultrasound characteristics of USL lesions were summerized and divided into four types according to morphological changes. The sensitivity, specificity, positive predictive value (PPV), negative predictive value (NPV), and accuracy of TVS for diagnosing USL endometriosis were also analyzed. Patients who were confirmed to have bilateral involvement while TVS diagnosis was unilaterally affected were classified as false negative cases.

## Results

### Surgical Findings

All 118 Patients were confirmed DIE by surgery and histopathology and their average age were 35.2 ± 6.2 years. In all cases, 85 had USL involvement, with 62 cases bilateral and 23 cases unilateral as shown in Table 1. For the cases with USL unilateral involvement, 15 were left-side and 8 were right-side. The other involved locations were intestines (60), pouch of Douglas (36), rectovaginal septum (28), bladder (14), vagina (9) and ureters (8), in descending order.

**Table 1:** Distribution of 85 cases of USL confirmed by surgery.

	Bilateral	Unilateral	
		Left	Right
N	62	15	8
%	73.0	17.6	9.4


*USL, uterosacral ligament involvement; N, number of patients; %, percentage*.

### TVS Findings

In all the 118 DIE patients, TVS found 84 patients with USL endometriosis and 81 of which were confirmed by surgery and histopathology. Of the 81 patients, 60 had bilateral involvement and 21 had unilaterally involvement (13 left, 8 right).

**FIGURE 1:**
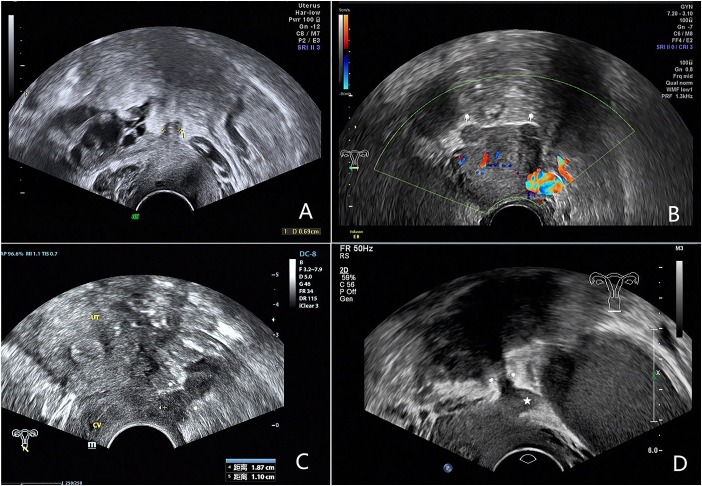
Images showing four types of USL lesions according to ultrasound characteristics of USL endometriosis. **(A)** Type I: the root segament of USL is thickened and stiff. **(B)** Type II: the arrows show endometriosis nodules on both sides of the USL. **(C)** Type III: the arrows show hypoechogenic irregular striped endometriosis lesion. **(D)** Type IV: the arrows show irregular striped endometriosis lesion of the right USL, the Pentagram shows a nodule of the left USL.

We analyzed the ultrasound features of 81 confirmed cases and summerized 4 types of USL lesions: type I. thickened and stiff lesions (Figure 1A), in this type the root segment (the insertion of the ligament on the cervix) of USL was stiff, thickened and hypoechogenic and the middle and posterior portions were not visible; type II. local nodules (Figure 1B), which were visualized as local round or stellate hypoechogenic lesions with regular or irregular margins; type III. irregular striped lesions (Figure 1C), the lesions distribute along USL or adhered into other organs and they were generally larger than type I and II, the root and middle part or even posterior part of USL were usually involved; type IV. mixed lesions (Figure 1D), the lesions involved both sides and have two types of the above.

Table 2 shows there were 10 type I, 6 type II, and 5 type III lesions in patients with unilateral involvement and 21 type I, 19 type II, 15 type III, and 5 type IV lesions in patients with bilateral involvement.

**Table 2:** Distribution and type of USL involvement in 81 confirmed cases.

	Bilateral	Unilateral	
		Left	Right
N	60	13	8
%	74.1	16.0	9.9
I	21	6	4
II	19	4	2
III	15	3	2
IV	5		


*USL, uterosacral ligament; TVS, transvaginal sonography; %, percentage*.

### Value of TVS for the Diagnosis of USL Endometriosis

The Sensitivity, Specificity, PPV, NPV, and accuracy of TVS for the diagnosis of USL endometriosis were 95.3, 90.9, 96.4, 88.2, and 94.1%, respectively, as shown in Table 3.

**Table 3:** The value of TVS in diagnosis of USL involvement in deep endometriosis.

Location	Sensitivity (%)	Specificity (%)	PPV (%)	NPV (%)	Accuracy (%)
USL	95.3	90.9	96.4	88.2	94.1
	(81/85)	(30/33)	(81/84)	(30/34)	(111/118)


*TVS, transvaginal sonography; USL, uterosacral ligament; NPV, negative predictive value; PPV, positive predictive value.*

There were 4 false negative and 3 false positive cases. Of the 4 false negative patients, 2 were found to be unilaterally involved by TVS, but actually they were bilaterally affected. The other 2 patients were missed diagnosis because they had multiple lesions adhered together in the retrocervical region. Of the 3 false positive cases, one patient was diagnosed as USL tumor by histological examination and 2 patients were confirmed as inflammatory lesions.

## Discussion

Diagnosing USL endometriosis with TVS has always been a difficult point in clinical research. Diagnostic rates varied greatly due to differences in diagnostic methods and diagnostic experience. A recent meta-analysis (Guerriero et al., 2015) demonstrated that the overall pooled sensitivity of TVS diagnosis USL endometriosis was 53%. A series of two studies by Bazot et al. (2003, 2004) showed that the sensitivity and accuracy of TVS to diagnose USL endometriosis were around 70.6–75% and 77–83.8%, respectively. However, in this study, the sensitivity and accuracy of TVS for the diagnosis of USL endometriosis were 95.3 and 94.1%, respectively, which were higher than those reported in most similar studies.

**FIGURE 2:**
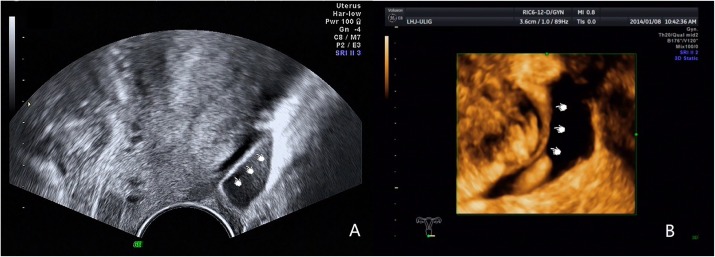
Images showing the normal USL imaged by 2D and 3D ultrasound in patients with fossa effusion. **(A)** Normal USL imaged by 2D ultrasound. **(B)** Normal USL imaged by 3D ultrasound.

We obtained such good results due to increased awareness of the disease and improved diagnostic methods. In clinical research we have found many factors could lead to low diagnostic rates. Firstly, the retrocervical area was a small and complex anatomical region, the borders of involved organs become indistinguishable in DIE, especially in patients with severe adhesions. Secondly, many examiners were not familiar with the normal ultrasound imaging of USL, which was crucial to improve the diagnostic rate. Some studies (Bazot et al., 2004; Exacoustos et al., 2017) mentioned that the USL was invisible using TVS. However, as early as Ohba et al. (1996) reported that transrectal ultrasonography can be used to observe the USL in non-endometriosis patients and in patients with endometriosis and that the thickness of the USL was associated with clinical symptoms. In our experience, the normal USL can be easily observed in patients with fossa effusion, and the root portion can be seen in a few patients in the absence of fossa effusion. Our experienced examiners can image normal USL using 2D and 3D ultrasound in patients with fossa effusion (Figure 2). Normal USL is visible as a nearly isoechoic arc from the upper cervix extending to the rectum. Lesions can be identified easily if sonographers can master the anatomy of the USL. Thirdly, the examiner’s experience was also an important factor affecting the accuracy. Researchers generally believed (Exacoustos et al., 2017) that the diagnosis of DIE required far more experience, and TVS was highly accurate for the noninvasive diagnosis of DIE in well-trained staff (Guerriero et al., 2015; Tammaa et al., 2015). The examiner of this study was experienced and professionally trained. At last, supplementary methods such as “tenderness-guided” methods and the use of a stand-off to visualize the near-field area (Guerriero et al., 2007) were also important to improve the detection rate. In our experience, the diagnostic accuracy will be lower if examiners do not pay attention to these factors.

In terms of classification of USL lesions, we summerized four types according to ultrasound characteristics. The purpose of our classification was to improve the examiner’s understanding of the disease and to quickly and accurately identify USL lesions. The results demonstrated that types I and II were very common, but type IV was rare among the four types. This study also concluded the detailed distribution of USL involvement according to surgical findings. The most commonly involved locations were bilateral, the left side and the right side in descending order. Similarly, Charles Chapron discovered that DIE was more likely to affect the left USL than the right side (Chapro et al., 2001). However, several previous studies (Jenkins et al., 1986; Fabio and Ginecologica, 1994) failed to find any significant asymmetry in the location of endometriosis involving the USLs. The reasons for the high incidence of bilateral involvement in this study may be that most of the patients had severe endometriosis, and pelvic involvement was more serious. Of course, our conclusions also need support from a larger sample of clinical statistics.

In addition, we noted that the specificity (90.9%) of TVS in diagnosis of USL endometriosis was relatively lower in our study. Bazot et al. (2004) and Guerriero et al. (2015) reported the specificity of 95.9 and 93%, respectively. There may be two reasons for this difference. Firstly, in our statistical analysis, we classified two patients (who were diagnosed unilateral lesion by TVS, but diagnosed as bilateral involvement during surgery) as false negative cases. However, this situation was not elaborated in other studies. Secondly, the other two cases had multiple lesions and severe adhesion in the posterior of the cervix which lead to difficulty in analysis. In this study, there were also three false positive cases. One patient was confirmed as a tumor by histology and another two patients were confirmed as inflammatory lesions. Therefore, it is also important to note that not all USL lesions are endometriosis, and a differential diagnosis should be made to rule out tumors and inflammatory changes (Nascu et al., 2006).

Some studies (Abrao et al., 2007; Bazot et al., 2009) reported that TVS has a lower sensitivity and accuracy for diagnosing USL involvement than MRI. However, our study showed that TVS has great value in the preoperative diagnosis of USL involvement with a sensitivity and accuracy of 95.3 and 94.1%, respectively. In addition, TVS is cost-effective, well accepted and widely available compared with MRI, so TVS should be used as the first line diagnostic technique (Saccardi et al., 2012).

### Limitation

The present study does have some limitations. Firstly, only patients with severe pelvic endometriosis and surgical evidence of DIE were included which may increase the diagnostic rate. Secondly, we summarized the ultrasound characteristics of USL involvement in the study into four types and whether there are other types should be further studied.

## Conclusion

Four types of USL endometriosis were summarized according to the ultrasound characteristics and the distribution of USL involvement was also concluded according to the results of surgery. TVS has significant value in diagnosis of USL endometriosis and can be used as a first-line tool for diagnosis.

## Ethics Statement

The Medical Science Ethics Committee of Shenzhen People’s Hospital approved this study (No. 2018100). Each patient or an appropriate family member provided informed written consent to obtain clinical materials.

## Author Contributions

YZ and BD conceived and designed the whole experiments and drafted the manuscript. JX performed the statistical analysis and interpreted the data. QL contributed to the clinical examination. XX contributed to the literature research. FX acquired the data. All authors read and approved the final manuscript.

## Conflict of Interest Statement

The authors declare that the research was conducted in the absence of any commercial or financial relationships that could be construed as a potential conflict of interest.
